# Warm climates of the past—a lesson for the future?

**DOI:** 10.1098/rsta.2013.0146

**Published:** 2013-10-28

**Authors:** D. J. Lunt, H. Elderfield, R. Pancost, A. Ridgwell, G. L. Foster, A. Haywood, J. Kiehl, N. Sagoo, C. Shields, E. J. Stone, P. Valdes

**Affiliations:** 1 Cabot Institute, and School of Geographical Sciences, University of Bristol, University Road, Bristol BS8 1SS, UK; 2 Department of Earth Sciences, University of Cambridge, Downing Street, Cambridge CB2 3EQ, UK; 3 Cabot Institute, and School of Chemistry, University of Bristol, Cantock's Close, Bristol BS8 1TS, UK; 4 Ocean and Earth Science, University of Southampton, European Way, Southampton SO14 3ZH, UK; 5 School of Earth and Environment, University of Leeds, Woodhouse Lane, Leeds LS2 9JT, UK; 6 Climate and Global Dynamics, National Center for Atmospheric Research, 1850 Table Mesa Drive, Boulder, CO 80305, USA

**Keywords:** palaeoclimate, future climate, modelling, proxy data

## Abstract

This Discussion Meeting Issue of the *Philosophical Transactions A* had its genesis in a Discussion Meeting of the Royal Society which took place on 10–11 October 2011. The Discussion Meeting, entitled ‘Warm climates of the past: a lesson for the future?’, brought together 16 eminent international speakers from the field of palaeoclimate, and was attended by over 280 scientists and members of the public. Many of the speakers have contributed to the papers compiled in this Discussion Meeting Issue. The papers summarize the talks at the meeting, and present further or related work. This Discussion Meeting Issue asks to what extent information gleaned from the study of past climates can aid our understanding of future climate change. Climate change is currently an issue at the forefront of environmental science, and also has important sociological and political implications. Most future predictions are carried out by complex numerical models; however, these models cannot be rigorously tested for scenarios outside of the modern, without making use of past climate data. Furthermore, past climate data can inform our understanding of how the Earth system operates, and can provide important contextual information related to environmental change. All past time periods can be useful in this context; here, we focus on past climates that were warmer than the modern climate, as these are likely to be the most similar to the future. This introductory paper is not meant as a comprehensive overview of all work in this field. Instead, it gives an introduction to the important issues therein, using the papers in this Discussion Meeting Issue, and other works from all the Discussion Meeting speakers, as exemplars of the various ways in which past climates can inform projections of future climate. Furthermore, we present new work that uses a palaeo constraint to quantitatively inform projections of future equilibrium ice sheet change.

## 1. Introduction

**Figure 1.**
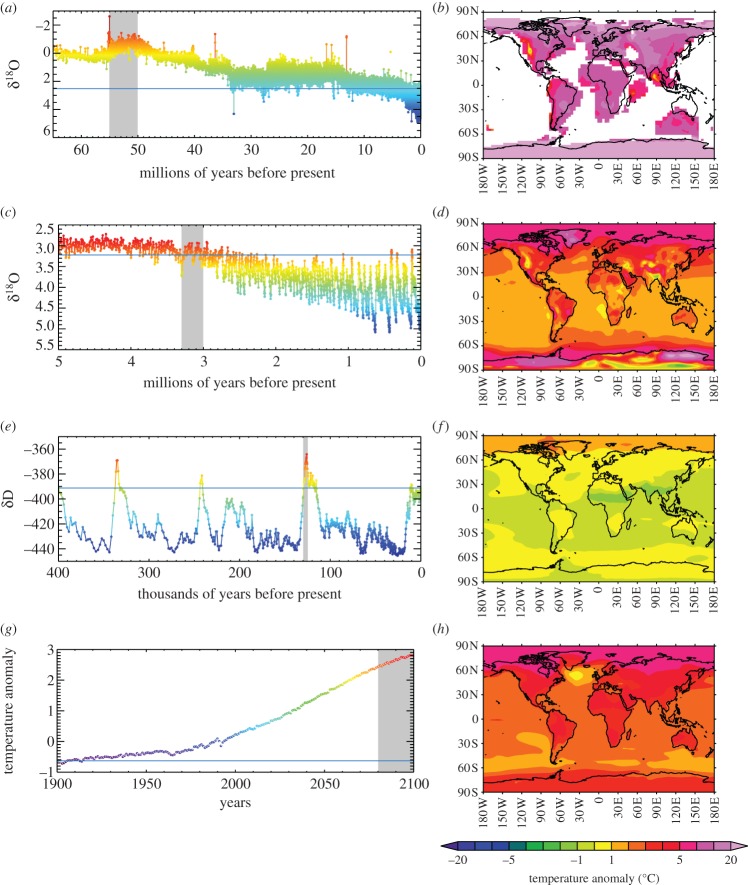
Warm periods of the past and future, as indicated by past climate data and models. (*a*) Benthic δ^18^O record of Cramer *et al.* [1], shown from 65 Ma to the modern. The grey highlighted period is the early Eocene (55–50 Ma). The blue horizontal line is an approximation to the pre-industrial value. The colours are a qualitative indication of temperature, going from colder (blue) to warmer (red). (*b*) Early Eocene annual mean continental temperatures relative to pre-industrial from the EoMIP model ensemble mean [2]. (*c*) Benthic δ^18^O record of Lisiecki & Raymo [3], shown from 5 Ma to the modern. The grey highlighted period is the mid-Pliocene (3.3–3 Ma). The blue horizontal line is an approximation to the pre-industrial value. The colours are a qualitative indication of temperature, going from colder (blue) to warmer (red). (*d*) Mid-Pliocene annual mean surface air temperatures relative to pre-industrial from the PlioMIP model ensemble mean [4]. (*e*) Ice core δD record of EPICA Community Members [5], shown from 400 ka to the modern. The grey highlighted period is the early Last Interglacial (LIG; 130–125 ka). The blue horizontal line is an approximation to the pre-industrial value. The colours are a qualitative indication of temperature, going from colder (blue) to warmer (red). (*f*) Early LIG annual mean surface air temperatures relative to pre-industrial from the LIGMIP model ensemble mean [6]. (*g*) CMIP3 model ensemble near-surface global mean temperature evolution for the A1B emissions scenario [7]. The grey highlighted area is the end of this century (2070–2100). The blue horizontal line is an approximation to the pre-industrial value. The colours are a qualitative indication of temperature, going from colder (blue) to warmer (red). (*h*) CMIP3 model ensemble near-surface global mean temperature in 2070–2100 minus 1900–1930 for the A1B scenario (data downloaded from the KNMI Climate Explorer, http://climexp.knmi.nl).

A central tenet of geology is the uniformitarian principle, which can be summarized as ‘the present is the key to the past’. Here, we ask to what extent ‘the past is the key to the future’. There are various ways in which past climates can inform future climate projections. Most broadly, information can be gleaned either from palaeo data (e.g. reconstructions of past climates derived from the geological record), or from a combination of numerical models of the Earth system and palaeo data. It is rare that numerical models alone can inform our understanding of the relationship between past and future climates—this work will always be underpinned by palaeo data either in terms of the boundary conditions prescribed in a numerical model, or by model–data comparison. A further distinction is between qualitative or contextual information, compared with quantitative information. In addition, in certain instances, the palaeo record can potentially provide a partial analogue of equilibrium future climate change. Here, we discuss these various aspects, in turn, drawing on examples from this Discussion Meeting Issue and other works from the Discussion Meeting speakers, as well as presenting some new work; the examples come from several warm periods from the past approximately 100 Myr, which themselves span periods of between a few thousand years and several million years. Figure 1 shows three key past climate records (figure 1*a*–*f*) that illustrate some of these warm periods in the context of global environmental change over a range of temporal scales, and compares them with future predictions (figure 1*g*,*h*).

It should be noted that, although such applications of past climate research are very important, probably the strongest motivation for the science presented in the papers in this Discussion Meeting Issue is a desire to understand the world we live in, and the complex and fascinating processes that have controlled its evolution over millions of years.

## 2. Qualitative information from data

Palaeo data can provide qualitative indicators of possible future climate evolution, and can put recent and future climate changes into context. Such palaeo data can often directly record the environmental characteristics of a past time period. An example is the presence of fossilized leaves in Antarctic sediments dated at approximately 100–50 Ma [8]. Over these time scales, plate tectonics can shift continental positions substantially, but Antarctica has remained situated over the South Pole for all of this time; as such, this provides direct evidence that the Earth can exist in a state that is very different from that of the modern day or the recent past, with a reduced Antarctic ice sheet, and polar regions warm enough to sustain ecosystems that are seen today in more equatorward regions. Other fossilized remains of vegetation and pollen—for example, a recent compilation of the early Eocene (approx. 50 Ma) by Huber & Caballero [9]—imply warming in mid-latitudes, and even more pronounced warming towards the poles, indicating a reduced pole-to-equator temperature gradient during warm climates.

We also have evidence that these warm periods were associated with high concentrations of atmospheric CO_2_ (e.g. during the early Eocene, approx. 50 Ma, Beerling & Royer [10] show a ‘best’ estimate of approximately 1000 ppmv); taken along with our understanding of the physics of the atmosphere and radiation and the greenhouse effect, this is consistent with the idea that increases in CO_2_ can have a large influence on the Earth system. However, without well-constrained proxy evidence for exactly how much higher CO_2_ concentrations were, this information is only qualitative, and so cannot tightly constrain the *sensitivity* of climate to changes in atmospheric CO_2_, i.e. the amount of warming for a given CO_2_ or other forcing change. Furthermore, it is possible that the warmth and reduced latitudinal temperature gradient, at this time, was caused not only by elevated CO_2_ but also by other forcings, for example, continental configuration and mountain height [11,12], or the lack of Antarctic ice [13] due to changes in the connectivity of large ocean basins through the opening or constricting of seaways and straits [14]. Nonetheless, modelling work does indicate that for major climate transitions, for example those associated with the inception of Antarctic and Northern Hemisphere glaciation, it is the CO_2_ forcing that dominates over direct tectonic forcings [15,16].

Moving to more recent warm time periods, the early and mid-Holocene (9–6 ka) provide more evidence that the Earth system can enter unusual states—lake-level and pollen data suggest that, during this period, regions of the Sahara were vegetated [17], and lakes covered much of the land surface in this region [18]. This is thought to be driven largely by changes in the Earth's orbit and angle of rotation [19]. The temperature changes induced by these astronomical drivers are similar in magnitude to those expected in the next century due to increasing atmospheric concentrations of CO_2_ [20]. The Last Interglacial (LIG; approx. 130–125 ka) provides another instance of unusual climate states driven by changes in the Earth's orbit, in this instance, leading to a reduction in the extent and volume of the Greenland ice sheet [21] and higher global sea levels [22,23] compared with modern.

In summary, qualitative palaeoclimate data indicate that Earth's climate and environment can change significantly due to natural drivers: temperatures vary by several degrees, vegetation patterns shift and evolve, and ice sheets wax and wane resulting in sea-level falls and rises. The key aspect is that the data tell us about a state of the Earth that actually existed in reality—not a construct of a numerical model, but something that is tangible and real.

## 3. Quantitative information from data

Many of the chemical or biological properties of past sediments, or the flora or fauna within them, have been calibrated to specific climate variables (e.g. temperature or precipitation) in the modern world, such that their determination in the past, combined with our best understanding of the physical systems that control these relationships, can be used to quantitatively evaluate the ancient climate state. Such climatic information is most relevant to our understanding of the future when the forcing that caused the inferred climate state can also be quantified, as this allows the sensitivity of the system to be estimated. For example, quantification of past CO_2_ levels and global temperature allows us to estimate the sensitivity of the climate system to a CO_2_ forcing (if it is assumed that it is the CO_2_ that is the primary driver of the temperature change). Considerable challenges are associated with estimating both the forcing and the response.

For periods older than approximately 3 Ma, the temperature signals of warm climates are relatively large, and, despite uncertainties in temperature proxies, in some instances, have a large signal-to-noise ratio. A dataset that has been developed with the purpose of providing a synthesis of past temperature data is presented by Dowsett *et al.* [24], who describe a vision for ‘PRISM4’—the next generation of global temperature database for the mid-Pliocene warm period (approx. 3 Ma), including, critically, assessment of confidence in all the proxies.

For these older time periods, climate change is thought to have been primarily driven by changes in atmospheric greenhouse gases. However, the proxies for climate forcing (primarily CO_2_ proxies) themselves have large uncertainties, and the influence of plate tectonics is not negligible, so it is most probably the forcing term that introduces most uncertainty into estimates of sensitivity (although it should be noted that there is agreement among all the proxies that CO_2_ was significantly higher than in pre-industrial periods during the greenhouse climates of the Eocene). Two papers in this volume aim to characterize the signal and uncertainties in CO_2_ proxies from past warm climates. First, Zhang *et al.* [25] produce a new record of CO_2_ for the past 40 Myr, making use of the alkenone proxy; these data reveal larger CO_2_ changes during key transitions in climate state than has previously been reconstructed using this proxy. Second, Badger *et al.* [26] focus on the time period 3.3–2.8 Ma, just before the expansion of Northern Hemisphere glaciation. They show a relatively stable CO_2_ signal during this time period, in contrast to previous work [27]. This stability in forcing reflects relatively stable global temperature indicators during the same interval [3].

The relationship between the forcing and the response of the Earth system is commonly expressed in the important metric ‘climate sensitivity’. This can be defined in several ways, for example the global annual mean near-surface (1.5 m) air temperature (SAT) equilibrium response due to a doubling of atmospheric CO_2_ concentrations, or more generally as the SAT response to a prescribed radiative forcing, usually 1 or 4 W m^−2^ (4 W m^−2^ is close to the radiative forcing for a doubling of CO_2_, but has the advantage that the forcing is model-independent). Furthermore, climate sensitivity can be defined to include long-term feedbacks related to slow processes such as ice sheets and vegetation (the ‘Earth system’ sensitivity), or just those processes which adjust on the time scale of decades, such as clouds, snow and sea ice (‘fast feedback’ or ‘Charney’ sensitivity). Because of the importance of this metric for characterizing future warming, the palaeo community has increasingly made efforts to constrain it from both palaeo data and models. Hansen *et al.* [28] use palaeo data to evaluate the SAT response to a CO_2_ forcing, using data from the past 40 Myr. They estimate the forcing from palaeo CO_2_ proxies, and the global mean response from the ratio of oxygen isotopes (δ^18^O) in deep-ocean-dwelling fossils from ocean sediments. By taking account of the component of change due to the slower varying ice sheets, they interpret the results as indicating a ‘fast feedback’ climate sensitivity of 4^°^C for a CO_2_ doubling. Using deep-ocean temperatures as opposed to sea surface temperatures (SSTs) directly has the advantage that the deep-ocean temperature is much more spatially homogeneous than the surface temperature, meaning that a relatively small number of sites can be used to robustly estimate the global mean. However, uncertainties in these estimates include the conversion factor from δ^18^O to SAT, and in particular how this has varied with climate state. Another approach is to use proxies for SAT or SST directly, but this has the disadvantage that a relatively large number of sites are needed to robustly estimate the global annual mean, and there still remains some uncertainty in the conversion from SST to SAT, as well as the uncertainties inherent in the SST and SAT proxies themselves. Instead of estimating the global mean response, this approach may be more suited to estimating a regional temperature response, which is calculated only in those regions of high spatial data density. There are also possibilities to reconstruct variables other than temperature, for example using vegetation data to estimate changes in the hydrological cycle.

When comparing palaeo-data-derived estimates of climate sensitivity (whether sensitivity to CO_2_, or any forcing) with future climate sensitivities from models, it is critical to ensure that a consistent comparison is being made. Most future climate sensitivity estimates from models only include ‘fast’ feedbacks in the climate system, and so produce estimates of future Charney sensitivity. However, the real world always responds with all feedbacks, both fast and slow, and so palaeo-derived estimates will include a fraction of these feedbacks, depending on the time scales over which the palaeo data are derived. Over very long time scales, all feedbacks will respond and so long-term data inform us about the Earth system sensitivity, which is generally higher than Charney sensitivity. It is possible to estimate the effect of long-term feedbacks, and therefore convert data-derived Earth system sensitivity estimates into Charney sensitivity estimates, in order to more readily compare palaeo data with models. A framework for achieving this has been recently suggested by Rohling *et al.* [29], who highlight the importance of consistently defining processes as either forcings or feedbacks. An alternative is to take the opposite approach, and include these long-term feedbacks into models, so that they are more directly comparable with the proxy data; these long-term feedbacks can themselves be estimated using palaeo proxy data [4,30], see §6).

However, the question remains: Even if we could exactly estimate climate sensitivity from palaeo data, what is the relationship between past climate sensitivity and future sensitivity? Climate sensitivity is likely to be dependent on the background state [31]. For example, if the Earth system is close to a threshold, then a relatively small forcing will result in a large response, an issue discussed by Hansen *et al.* [28]. Examples include the Eocene–Oligocene boundary, when the Earth cooled enough to support extensive ice on Antarctica, and additional cooling was amplified by ice sheet feedbacks; or the last deglaciation, approximately 15–10 ka, when large ice sheets were melting and providing additional feedbacks to global warming. Such past time periods, close to thresholds, may be unsuitable for estimating future sensitivity, although various climate ‘tipping points’ may be crossed in the future [32]. Furthermore, very warm periods such as the Cretaceous or early Eocene may be less relevant, due to the likely lack of cryospheric feedbacks, and/or differing properties and behaviour of clouds [33].

In the relatively recent periods of the past approximately 1 Myr, the main climate forcings are normally well constrained, either by astronomical theory (with the forcing known accurately back to about 50 Ma [34]), or by greenhouse gas concentrations derived from ice cores [35]. However, compared with more ancient climates, the warm periods during this period are relatively similar to the pre-industrial Earth, and so the challenge is to robustly reconstruct a relatively small temperature signal (i.e. the response), given the uncertainties in the temperature proxies. Some progress has recently been made in this field, with data syntheses for the LIG warm period (130–125 ka) being presented by Turney & Jones [36] and McKay *et al.* [37], and for the mid-Holocene by Bartlein *et al.* [17]. However, although the astronomical forcing is well known for these time periods, a simple metric for defining sensitivity to this forcing has not been defined. This is because the forcing has a complex seasonal and latitudinal structure, and is close to zero on the annual global mean. As such, the response of the system to this strongly seasonal and regional forcing cannot be directly extrapolated to infer a sensitivity to future CO_2_ forcing.

Of course, a change in temperature is not the only lesson for the future from past warm intervals: it is likely that many aspects of the Earth system—including precipitation, ice volume and sea level, and seasonality—also changed. Other work presented in this Discussion Meeting Issue [38] provides stimulating evidence that even fundamental aspects of the Earth's carbon cycle could have differed in a warm Earth; in particular, the authors suggest that removal of carbon from the atmosphere and surface ocean would have been inhibited in warm oceans where organic matter is more effectively respired.

## 4. Qualitative information from model–data comparisons

Probably the most common way that palaeo data and palaeo models come together to inform future predictions is in the form of model–data comparisons. Model predictions of the future cannot be tested directly with data. However, some confidence can be gained in future model predictions if, when configured for simulating a past climate, the model produces results that are in agreement with palaeo data. Similarly, future predictions from models that do not perform well for past climates may be viewed with caution. This has been discussed in the context of using the relatively warm mid-Holocene, providing possible constraints on future El Niño Southern Oscillation (ENSO) variations [39].

When models produce results that are inconsistent with reconstructed proxy data for past climates, this can be due to one or more of three possibilities: (i) the model has a fundamental misrepresentation of physical or dynamical Earth system processes; (ii) the model has been given the wrong forcing; (iii) the palaeo proxy data have been misinterpreted. When confronted with poor model–data comparisons, scientists have to make reasoned decisions about which of these possibilities is the most likely, and either modify the model, carry out simulations with new forcings, or reinterpret the data, or a combination of all three. If the model–data comparison improves, then more confidence is gained in the model future predictions. So, although the model–data comparison itself is likely to be quantitative in its methodology, the implications for future climate are largely qualitative.

**Figure 2.**
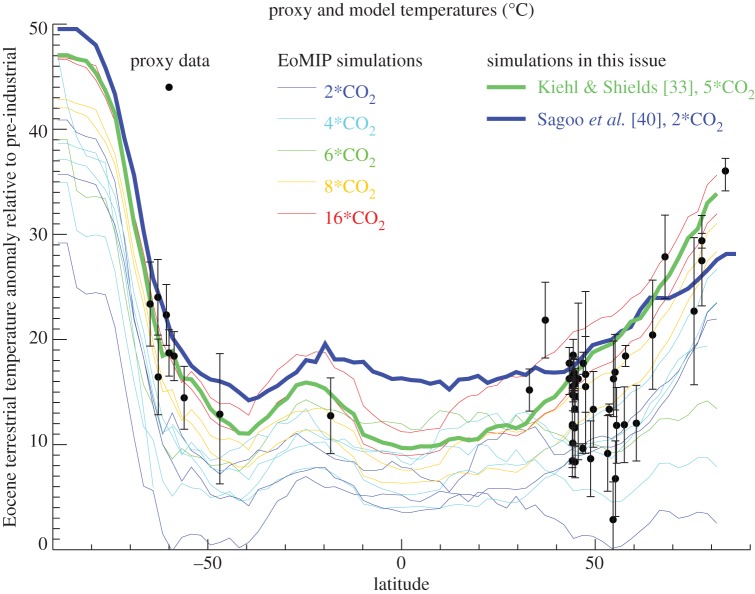
Comparison of early Eocene modelled surface air temperature (SAT) warming relative to pre-industrial, with proxy-derived temperatures, *Δ*SAT versus latitude. For the model results, the solid lines represent the Eocene continental zonal mean minus the pre-industrial global zonal mean, with the colour indicating the CO_2_ level at which the simulation was carried out. Thin lines represent those EoMIP models compiled in Lunt *et al.* [2], and the thicker lines represent the Kiehl and Shields [33] and Sagoo *et al.* [40] simulations from this Discussion Meeting Issue. For the proxy data, the symbols represent the proxy temperature, and the error bars represent the range, as given by Huber & Caballero [9].

This approach is taken by three papers in this volume. The first focuses on the LIG (approx. 130–125 ka). Otto-Bliesner *et al.* [20] carry out simulations of the LIG with a climate model developed at the US National Center for Atmospheric Research, CCSM3, forcing the model with the orbital configuration of that time, and greenhouse gases as recorded in Antarctic ice cores. They find a relatively poor model–data agreement in terms of the modelled SSTs. They then go on to explore some reasons for this, and carry out additional simulations in which the West Antarctic ice sheet is reduced. This marginally improves the model–data comparison, but they also question the extent to which proxies may be systematically biased towards specific seasons. Kiehl & Shields [33] and Sagoo *et al.* [40] both address a long-standing problem in palaeoclimate model–data comparisons—that models do not, in general, simulate polar regions of the Early Eocene that are as warm as indicated by proxy temperature data when given CO_2_ forcings that are within the uncertainties of proxy CO_2_ data. By modifying the properties of clouds in their model, Kiehl and Shields [33] test the hypothesis that this is due to the treatment of aerosols in models, and in particular that the effect of aerosols on cloud formation and development assumes implicitly a modern aerosol distribution. They find that the model–data comparison greatly improves when the aerosol assumptions are modified. Sagoo *et al.* [40] take a different approach—they modify several ‘tuneable’ parameters in their climate model, producing an ensemble of 115 simulations. They find that one of these ensemble members (member ‘E17’) produces results that simulate an Eocene climate in good agreement with the proxies, while also retaining a good modern simulation. This ensemble member produces a modern Charney climate sensitivity of approximately 3^°^C. These new simulations of Sagoo *et al.* [40] and Kiehl and Shields [33] are shown in figure 2, which also includes simulations conducted previously as part of the model-intercomparison project, EoMIP [2]. It is clear that these two studies can produce polar climates that are warmer for a given CO_2_ level compared with previous work, thereby significantly improving the model–data comparison when considering both CO_2_ and temperature data. For example, the r.m.s. error (calculated from a point-by point comparison of the palaeo data with the model temperature at the nearest gridbox) of the Sagoo *et al*. [40] simulation is 5.1^°^C, which should be compared with values from the previous EoMIP model simulations at the same CO_2_ concentration (560 ppmv, i.e. two times pre-industrial concentrations) of 15.5^°^C (HadCM3L model), 9.7^°^C (ECHAM model) and 11.5^°^C (CCSM3 model).

An additional qualitative use of model–data comparison is in the field of attribution. This is best illustrated using an example from the past millennium. Here, temperature data have been compiled to generate the well-known ‘hockey stick’ evolution of climate over the past 1000 years [41]. Model simulations of this time period can reproduce the observed temperature evolution well, when forced with reconstructions of the relevant drivers—greenhouse gases, land-use change, volcanic forcing and changes in solar output. However, when simulations are run without greenhouse gas forcing, the models agree well with the observed temperature changes up until the past approximately 150 years; at that point, they diverge, with the observed temperatures warming and the modelled temperatures staying relatively constant [42]. This implies that, if the correct forcings have been applied to the models, and the models are robust, then the recent warming is primarily due to increases in greenhouse gases.

## 5. Quantitative information from model–data comparisons

It is possible for model–data comparisons to provide more quantitative constraints on future climate change. This can be carried out in a Bayesian framework, where the palaeo model–data comparison is used to weight different instances of the model according to their fit to palaeo data, and/or rule out others, and use this information to weight the corresponding future projection. This has been carried out in the context of the Last Glacial Maximum (LGM) by Hargreaves *et al.* [43], who showed that using observations of LGM tropical temperatures allowed the equilibrium future climate sensitivity to be estimated as 2.5^°^C, with a high probability of being under 4^°^C. However, the utility of the mid-Holocene warm period for quantitatively constraining future projections has recently been questioned [44], owing to the relatively small signal-to-noise ratio at this time. The approach of weighting model simulations of the future according to their performance relative to past observations was used by Robinson *et al.* [45], who produced an ensemble of future ice sheet simulations, all of which were consistent with data from the LIG, the warmest period of the past 150 000 years.

**Figure 3.**
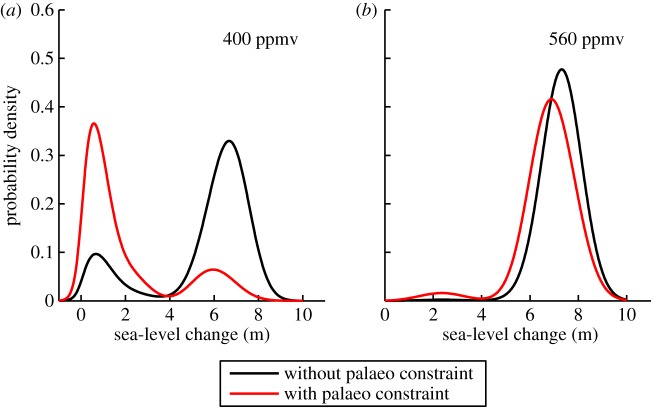
Probability density functions of future equilibrium contribution to sea-level rise from the Greenland ice sheet, under equilibrium CO_2_ scenarios of (*a*) 400 ppmv and (*b*) 560 ppmv. In each case, one PDF does not include a constraint based on palaeoclimate data (black line, without palaeo constraint) and the other (red line, with palaeo constraint) does. The simulations are carried out using the methodology presented in Stone *et al.* [46].

Here, we present a new analysis, similar to that of Robinson *et al.* [45], using a Bayesian approach to infer the future equilibrium volume of the Greenland ice sheet, and taking into account constraints from ice core data from the LIG. We extend the methodology presented in Stone *et al.* [46] (henceforth S13), by applying it to the future in addition to the past. S13 used a set of pre-industrial and LIG climate model simulations (HadCM3 [47]) to drive an ensemble (500 members) of ice sheet model (Glimmer [48]) simulations of the modern and LIG Greenland ice sheet. The ensemble of ice sheet models encompassed a range of values for five key parameters relating to the surface mass balance scheme, the dynamic flow of the ice, the ice sheet basal temperature and the atmospheric lapse rate. An efficient ‘pseudo-coupling’ methodology was devised to take account of the temperature elevation and the ice–albedo feedback, by calculating a climate forcing based on interpolation between climate model simulations, which included either a modern-day, partially melted or absent Greenland ice sheet, depending on the previous year's ice volume from the ice sheet model. In addition, the coupling took into account the temporal evolution of climate at this time by linearly interpolating between 130, 125 and 120 ka climates with different astronomical forcings. The modern (pre-industrial) Glimmer simulations were used to give each model instance a weighting based on its performance in terms of spatial ice thickness relative to ice thickness observations of the modern ice sheet. In addition, models were rejected if their LIG simulation did not produce ice at the site of the GRIP ice core, where data from ice cores indicate there was ice at this time. In the S13 work, these data were used in conjunction with Bayes' theorem to produce a probability density function (PDF) of LIG Greenland ice sheet volume (and hence sea-level contribution from the melted ice sheet), taking into account uncertainty in the ice thickness observation, and missing physical processes in the ice sheet model (for a more detailed description of the methodology, see S13). Here, we go one step further by carrying out future ice sheet simulations using the same pseudo-coupling methodology and PDF construction as described above, but with the ice sheet model driven by future climate scenarios (stabilization close to modern concentrations, 400 ppmv, and two times pre-industrial concentrations, 560 ppmv), for 50 000 years. The results are shown in figure 3: PDFs of future sea-level rise with either weightings based on the skill of the model for the modern alone, or with a weighting based on the skill of the model for the modern and the LIG data constraint. Figure 3*a* shows these two PDFs for the GrIS equilibrium state under a 400 ppmv climate. It can be seen that both PDFs are bimodal, resulting from the existence of two stable states in the ice sheet model; one where the GrIS only melts partially (around 1 m of sea-level rise) and another where almost complete melting occurs (around 7 m of sea-level rise). If the palaeo constraint is not included, then the PDF is skewed towards the higher melt state. If the palaeo constraint is included, the PDF is skewed towards the lower melt state. This implies that ignoring palaeo data, in this instance, would result in a prediction of the future equilibrium state of the ice sheet that was too extreme. Inclusion of the palaeo constraint under a 560 ppmv climate (figure 3*b*) has little influence over this future sea-level projection, which shows a probable high melt state with or without the palaeo constraint. Although the results themselves must be treated with great caution (due, for example, to physical processes that are missing from, or approximated in, the ice sheet model, and uncertainties associated with the climate model simulations that drive the ice sheet model), it does illustrate the potential of warm climates to inform future predictions in a quantitative way.

## 6. Partial analogues

The current rate of increase of CO_2_ emissions is unprecedented in the geological record; as such, there is no perfect analogue from the past for the temporal evolution of future climate [49]. However, in theory, it could be possible to find a past stable time period that was similar to the pre-industrial period but with elevated concentrations of atmospheric CO_2_. If such a period could be found, it could provide a partial analogue for a future equilibrium climate state, under an equilibrium CO_2_ concentration of the past time period (care should be taken in interpreting the analogue, because climate is a function not only of the forcing applied but also of the preceding climate, i.e. the initial condition). Such a time period would have to be in the past approximately 5 Myr, otherwise, the continental and seaway configurations may be too different from the modern to have direct relevance, and CO_2_ proxies become ever more uncertain. It also has to be older than 1 Myr, because the ice core record indicates that CO_2_ levels in this period never greatly exceeded pre-industrial values. Haywood *et al.* [50] identify such a time period—the KM5c period of the Piacenzian Stage of the Pliocene, about 3.3 Ma. At this time period, the continental configuration, topography and orbital configuration were close to those of the modern day, and many of the taxa existing then are currently extant. As such, this time period provides a possible partial analogue for future equilibrium warming, if CO_2_ levels at this time can be well constrained. Dowsett *et al.* [24] also highlight this period as a target for future palaeo-data acquisition.

Models can also make use of these partial analogue time periods. Current generations of models do not simulate well some long-time-scale processes in the Earth system. Examples are vegetation and ice sheets. These processes are problematic because they act on long time scales and so are not readily testable with the observational record, and there is a lack of understanding of the underlying mechanisms, and how to represent these in a numerical form (e.g. for ice sheets, the evolution of the grounding line). As such, model simulations of the long-term future are problematic because (i) computationally, it is not possible to run a latest-generation model to full equilibrium and (ii) long-term processes are not well represented. However, if information on these long-term processes and their effects can be gleaned from partial analogues in the palaeo record, and the resulting changes to boundary conditions implemented directly in a model, then these problems can be overcome. This approach has been used previously for the Pliocene [4,30], where it showed that including the long-term feedbacks of ice sheets and vegetation directly into a model as boundary conditions resulted in an increase in climate sensitivity of about 50%.

Other past time periods, while not being analogues in the strictest sense, can provide interesting points of comparison with the future. Zeebe & Zachos [51] examine the impacts on climate, ocean acidification and marine calcifying organisms of the carbon released during the Palaeocene–Eocene thermal maximum (PETM, approx. 55 Ma). They then compare this with the likely impacts of current and future anthropogenic carbon release. They conclude that the anthropogenic carbon input rate is most probably greater now than during the PETM, causing a more severe decline in ocean pH and saturation state.

## 7. Conclusions

Reconstructing and modelling past climates and using that to inform future predictions of climate change is challenging. Nonetheless, clear lessons have emerged, some of which are explored by the papers in this Discussion Meeting Issue. There is very strong evidence throughout Earth history that climate does vary markedly, and can do so rapidly across thresholds or when subjected to a particularly strong forcing. Quantifying the climate forcings and responses is more challenging. However, past CO_2_ and temperature records can be combined to produce constraints on climate sensitivity, providing full account is taken of uncertainties in the forcing and response, and assuming CO_2_ is the main driver of the temperature change. Synthesis of past environmental change can be used to evaluate numerical models. Inconsistencies between models and data have been the stimulus to reassess both the data (through better quantification of uncertainties) and the models (through exploration of model sensitivities and experimental design), a process that has led to improved agreement. Indeed, this model–data comparison may potentially be used to provide quantitative constraints on future climate predictions, through a Bayesian approach.

Although there has been recent initial progress in using data and/or modelling of past warm climates to inform future climate predictions, many challenges remain. These include (but are not limited to) improved understanding and development of palaeo CO_2_ proxies, larger model ensembles and more (and more diverse) data with good global coverage, and integration of past climate test cases into the development cycle of climate models.

Drawing on examples from this Discussion Meeting Issue, and from the work of all the speakers at the associated Discussion Meeting, we have provided a brief overview of the various ways in which past warm climates can provide information on future climate change, through the use of data and modelling approaches. We hope that the papers in this Discussion Meeting Issue stimulate future research in this exciting and important field.
